# PLATFORM at 90 days: Evaluating the clinical utility of FFRCT

**DOI:** 10.21542/gcsp.2016.22

**Published:** 2016-09-30

**Authors:** Ahmed Kharabish, Ahmed ElGuindy

**Affiliations:** Aswan Heart Center, Aswan, Egypt

## Abstract

The advancement that took place in assessing fractional flow reserve (FFR) using various competing modalities led to numerous research trials to evaluate the clinical impact of each. Among those trials is the recently published PLATFORM study. The data was designed to compare two clinical scenarios; a combination of computed fluid dynamics with computed tomographic angiography (CTA-guided strategy) in non-obstructive coronary artery disease (CAD) on one arm, compared to the standard practice representing the other clinical arm. The study’s results were evaluated for further evidence and clinical insights.

## Introduction

Functional imaging is frequently utilized to evaluate patients with intermediate pretest likelihood for coronary artery disease (CAD) with the aim of answering three critical questions: (a) does the patient have obstructive coronary artery disease; (b) what is the functional significance of independent lesions; and (c) what is the extent of ischemia (or area of myocardium at risk)? The answers to these questions not only aid in identifying which patient requires invasive coronary angiography, but also guides the interventionist in identifying individual lesions that would benefit from revascularization. On the other hand, standard CT coronary angiography is capable of defining coronary anatomy as well as the overall atherosclerotic burden (coronary calcium score), but lacks the capability to assess the functional significance of individual lesions which represents an important limitation in patients with moderate stenoses.

By combining computational fluid dynamics (CFD) with standard CT angiography images, FFRCT has recently been developed with the aim of providing a noninvasive clue to the functional significance of individual lesions detected on CT coronary angiography.^1^ To date, the only modality capable of providing both anatomical and functional significance data is invasive coronary angiography coupled with fractional flow reserve (FFR) measurement. The invasive nature of the latter procedure explains the evolving interest in FFRCT.

**Figure 1.**
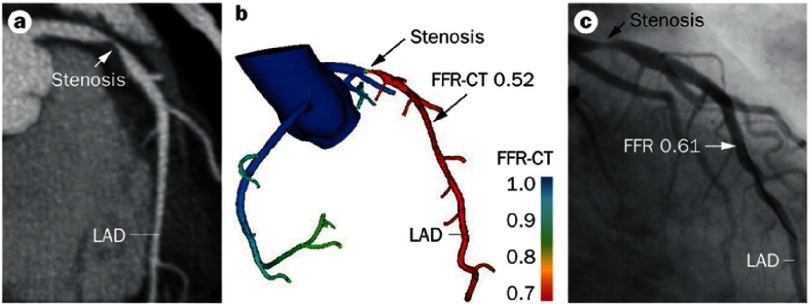
(a) Computed tomography (CT) images of a stenosis in the left anterior descending artery (LAD), (b) Fractional flow reserve (FFR) measured by CT and, (c) the corresponding images and FFR in invasive catheter.

FFRCT has recently been shown to have a good diagnostic accuracy compared to invasive FFR (Figure 1), with a sensitivity and specificity to detect FFR ≤ 0.8 of 86% and 79% respectively^2^.

However, the impact of FFRCT on clinical outcomes when incorporated into clinical practice remains largely unknown. The prospective longitudinal trial of FFRCT: outcome and resource impacts (PLATFORM) was designed to answer this question by testing the hypothesis that in patients with suspected stable coronary artery disease, a CTA-guided strategy would lead to fewer invasive angiograms with no obstructive CAD compared to standard practice, and would have low rates of major adverse clinical events. The study’s results were recently published in the European Heart Journal.^3^

## The study

PLATFORM was a multicenter, non-randomized, comparative study that prospectively enrolled 584 symptomatic patients without known CAD but with an intermediate pretest likelihood of obstructive CAD at 11 centers. All patients were ≥ 18 years old and were scheduled (by their treating physician) to have non-emergent, either non-invasive or invasive cardiovascular testing to evaluate suspected CAD. Patients with acute coronary syndromes, clinical instability, previously documented CAD, contraindications to CTA or requiring emergent or urgent procedures were excluded from the study.

**Figure 2.**
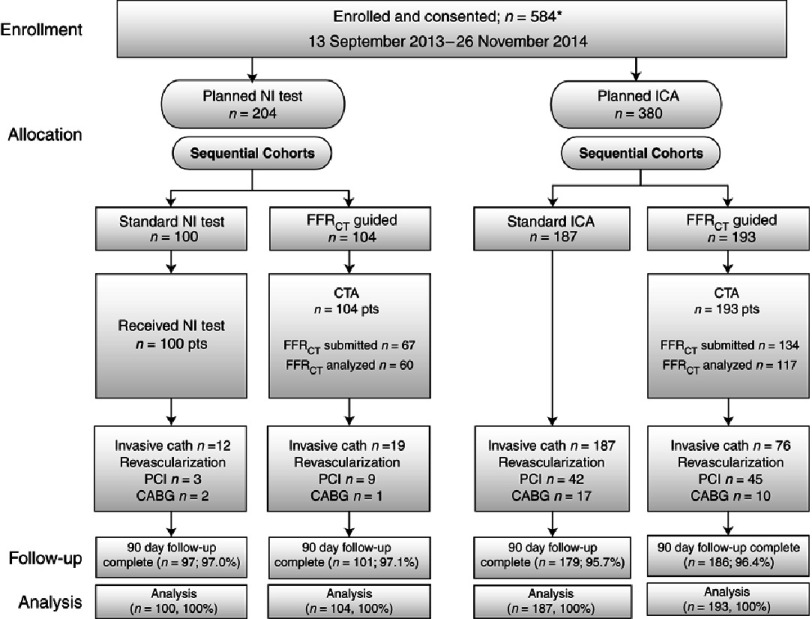
The initial study groups were categorized according to the planned investigational strategy and subsequent allocations. NI: noninvasive, ICA: invasive coronary angiography, FFRCT: fractional flow reserve computed tomography, CTA: CT angiography, PCI: percutaneous coronary angiography, CABG: coronary artery bypass grafting.

The study design was somewhat complex (Figure 2). Patients were enrolled in two consecutive cohorts: the first included patients planned for noninvasive imaging (NI) and the second included those planned for invasive coronary angiography (ICA). Both cohorts were then subdivided into usual care and FFRCT-guided strategy subgroups. FFRCT was performed when there was a ≥ 30% stenosis or if the patient was to be referred to ICA. A follow-up visit was performed at 90 days, 6 months, and 12 months from study entry. All CTAs were acquired using a ≥ 64-slice multi-detector single- or dual-source CT scanner.

Using the acquired CTA data, coronary blood flow was simulated under conditions that mimic intravenous adenosine to mirror pressure and flow data and the FFR numeric values that would have been obtained during an invasive evaluation. Analysis of the data was performed centrally using the HeartFlow software. FFR CT values in all vessels were scaled and color-coded.

The primary endpoint was the percentage of patients planned for ICA with no obstructive CAD and was compared between the usual care invasive testing vs. CTA/FFRCT-guided care arms. A comparison of the rate of ICAs with no obstructive CAD in those planned for NI testing was also carried out as a secondary endpoint. Obstructive disease was defined as either (i) an invasively measured FFR ≤ 0.80 in any segment, regardless of degree of stenosis, or (ii) QCA stenosis ≥50% in a vessel ≥2.0 mm diameter without an invasively measured FFR. The major safety endpoint was a composite of all-cause mortality, non-fatal myocardial infarction (MI), and unplanned hospitalization for chest pain leading to urgent revascularization at 90 days.

## Results

A total of 584 patients were enrolled for the study. 204 subjects were scheduled for a non-invasive test while 380 were scheduled for non-urgent invasive coronary angiography. 90-day data were obtained in 563 subjects.

100 patents were allocated to a standard NI test while 104 were studied by FFRCT. Only 67 FFCT studies were eventually submitted and analysis of the data was possible in only 60 patients due to problems with image quality or acquisition. The percentage of patients with no obstructive CAD on ICA did not differ between both arms. Patients in the FFRCT-guided arm were also exposed to significantly higher radiation doses compared to patients undergoing standard NI testing (8.8 ± 9.9 mSv vs. 5.8 ± 7.1 mSv respectively, p = 0.0002).

On the other hand, the 380 patients in the planned ICA arm were randomized to either ICA (n = 187) or to FFRCT-guided management (n = 193). 106 patients in the ICA subgroup (73.3%) were found to have no obstructive coronary disease compared to only 24 patients (12.3%) in the FFRCT-guided subgroup (p < 0.0001). Importantly, ICA was cancelled in 61% of patients after receiving FFRCT results. Amongst those who eventually underwent ICA (n = 76), 24 patients (31.6%) had no obstructive CAD. Radiation exposure did not differ between the ICA and the FFRCT subgroups. Only two major adverse cardiovascular events occurred in the planned ICA group who were assigned to FFRCT. One was a periprocedural MI in a subject whose CTA was of insufficient quality for FFRCT analysis, and the other was hospitalization for urgent revascularization following an FFRCT showing severe CAD. The authors concluded that CTA/FFRCT was a feasible and safe alternative to ICA and was associated with significantly lower rate of ICA showing no obstructive CAD.

## Discussion

The investigators are to be commended for conducting such a well-designed and complex study that reflects real-life clinical practice. FFRCT failed to show any incremental advantage over non-invasive testing in patients with stable CAD, with the disadvantage of exposure to higher radiation doses. On the other hand, the second arm of the study (planned ICA) suggests that FFRCT might be a reliable gatekeeper to ICA. While this conclusion is appealing, a number of factors make it questionable:

1. The study was not powered to detect differences in clinical outcomes. The real fate of patients in whom planned ICA was cancelled based upon FFRCT findings remains unknown as the study’s duration (90 days) is not sufficient to reliably establish the safety of such approach. Furthermore, with the low event rate observed in patients with stable coronary artery disease in general, it is unlikely that this can be resolved when the trial is concluded after 12 months.
2. 73% of patients in the ICA group were found to have no obstructive CAD. This strikingly high percentage strongly questions the true pretest likelihood of CAD in the study population.
3. Obstructive CAD was defined as either FFR < 0.8 or QCA showing >50% luminal stenosis. This might create the false impression that both parameters are interchangeable and/or carry the same prognostic value. In the landmark FAME trial, 65% of patients with angiographic stenoses of 50–70% were found to have an FFR value of more than 0.8.^4^
4. The study failed to demonstrate any advantage of FFRCT over other non-invasive tests as a gatekeeper to ICA. Based upon these findings, better adherence to practice guidelines that recommend non-invasive stress testing for the initial evaluation of patients with intermediate pretest likelihood of stable coronary artery disease^5,6^ might arguably obviate the need for another modality with questionable cost- and time-effectiveness compared to other well-established tests (although not without their own limitations).
5. Analysis of FFRCT was not possible in 21 patients out of 201 in whom in was requested. This relatively high rate (11.9%) of non-diagnostic tests suggests that this novel technology still requires further improvements before it is adopted in day-to-day clinical practice.

## What have we learned?

FFRCT combined with CTA is a novel modality that has the unique advantage of combining functional and anatomical evaluation of epicardial coronaries in one non-invasive test. Results from the PLATFORM study suggest a potential benefit of FFRCT in reducing the number of unnecessary invasive coronary angiography procedures. However, the clinical role of FFRCT and its added value compared to other non-invasive tests need to be evaluated in larger studies before it can be introduced to routine clinical practice.
